# Endothermy, neuron counts, and other issues: Further remarks on neurocognitive evolution in fossil vertebrates

**DOI:** 10.1002/ar.70113

**Published:** 2025-12-19

**Authors:** Kai R. Caspar, Cristián Gutiérrez‐Ibáñez, Hady George, Thomas R. Holtz, Darren Naish, Grant R. Hurlburt

**Affiliations:** ^1^ Institute of Cell Biology Heinrich Heine University Düsseldorf Düsseldorf Germany; ^2^ Department of Biological Sciences University of Alberta Edmonton Alberta Canada; ^3^ School of Earth Sciences University of Bristol Bristol UK; ^4^ Department of Geology University of Maryland College Park Maryland USA; ^5^ Department of Paleobiology National Museum of Natural History Washington, DC USA; ^6^ School of Biological Sciences, Faculty of Environment and Life Sciences University of Southampton Southampton UK; ^7^ Department of Natural History Royal Ontario Museum Toronto Ontario Canada

**Keywords:** comparative cognition, dinosaurs, endothermic brain hypothesis, neurology

## Abstract

Last year, we challenged the view that large‐bodied theropod dinosaurs such as *Tyrannosaurus rex* resembled primates in cognition and behavior, a proposition made by Herculano‐Houzel in 2023. More recently, Jensen et al. have criticized our work on this topic, raising methodological and conceptual issues. Central to their argument is the assumption that tachymetabolic endotherms should be expected to converge in neurocognitive traits, which follows the recently proposed endothermic brain hypothesis. We here respond to their critique, address critical misconceptions, and argue that none of the points raised by Jensen et al. challenge the conclusions we have drawn. We show that the endothermic brain hypothesis lacks robust support from the fossil record. As of now, no compelling evidence suggests that endothermy coevolved with enlarged brains or elevated neuron densities in either the avian or mammalian lineage. Various fossil groups containing endothermic taxa retain plesiomorphic endocast traits and do not converge with birds and mammals in the relative size and proportions of their brains. Furthermore, we elaborate on our discussion on (forebrain) neuron counts as correlates of cognitive performance and highlight that neuron numbers evolve in tandem with body mass in birds and mammals, suggesting that comparatively high neuron number estimates for some Mesozoic dinosaurs do not require explanations that orbit around exceptional cognitive abilities. Despite these disagreements, we identify significant overlap in opinion between Jensen et al. and ourselves, including in the position that neuron count estimates for Mesozoic dinosaurs will remain unreliable and are unsuitable for inferring cognitive complexity.

## 1 INTRODUCTION

Herculano‐Houzel's (2023) work on the application of endocast‐derived forebrain size and neuron count estimates to discussions of cognition in Mesozoic dinosaurs and pterosaurs has stirred both academic and popular debate. In the respective study, Herculano‐Houzel argues that absolute neuron numbers are useful proxies for the prediction of cognitive complexity and other biological traits; most notably, it includes the proposal that such large‐bodied theropods as *Tyrannosaurus rex* shared key cognitive features with anthropoid primates. In a previous study (Caspar et al., 2024), we criticized Herculano‐Houzel's approach and challenged her inferences, pointing to issues in her methodology and interpretations. We rejected the notion that Mesozoic dinosaurs were similar to modern primates in the relevant behaviors and, in addition, argued that telencephalic neuron count estimates are not helpful proxies for the modeling of cognition in these animals. Rather than relying on such neurological measures when considering cognition in fossil lineages, we called for integrative approaches while also highlighting their current limitations.

Jensen et al. (2025) entered the discussion more recently and criticized our work, claiming that it showcases inappropriate methods, insufficient expertise, and a lack of nuance regarding our line of argumentation and use of terminology. They suggest that the recently formulated endothermic brain hypothesis (EBH, Osvath et al., 2024) is key in understanding cognitive evolution in non‐bird dinosaurs, the ramifications of which were apparently overlooked by Caspar et al. (2024). While Jensen et al. (2025) appear to agree with Herculano‐Houzel (2023) on the usefulness of total telencephalic neuron counts as cognitive predictors, they echo and expand upon Caspar et al. (2024) in arguing that reliable neuron count estimates for Mesozoic dinosaurs cannot be obtained since both their neuron density and brain sizes cannot be inferred with the necessary precision. A point framed as the central insight of their article is their comment that the cognition of “*T. rex* was *T. rex*‐like,” a self‐evident statement that neither challenges the work of Herculano‐Houzel (2023) nor Caspar et al. (2024) and which does not warrant further discussion.

We find the commentary by Jensen et al. (2025) to include a number of misconceptions and distortions with regard to our work that we consider problematic. Here, we focus on core disagreements, our aim being to provide clarifications and additional arguments relevant to our previous article. We end with a summary of positions we share with Jensen et al. (2025), and highlight the fact that broad agreement exists with respect to several key aspects of this debate.

## 2 CLARIFICATIONS ON TERMINOLOGY AND CONTENT IN CASPAR ET AL. (2024)

We agree with Jensen et al. (2025) that the language often used to discuss cognition, be it in the academic or public sphere, is imprecise and, furthermore, that mention of Mesozoic dinosaurs exhibiting “primate‐like” or “crocodile‐like” cognition requires qualification. We concede that Caspar et al. (2024) should have been clearer in how they defined and used such terms and welcome the more extensive discussion provided by Jensen et al. (2025). However, we must also note that definitions applied to the term “cognition”—and assumptions about the phenomena it encompasses—are notorious for being subject to varied opinions (e.g., Bayne et al., 2019; Dvořáček & Kodrík, 2024). Caspar et al. (2024) did not consider the low‐level sensory processing underlying perception (such as that concerning neural activity in the olfactory bulbs, which we excluded from our brain volume estimates) as a cognitive phenomenon. In contrast, Jensen et al. (2025) argued for a more holistic interpretation of cognition that includes brain activity more concerned with initial processing of sensory input (“vision is cognition”). Adopting one or the other view within the dynamic spectrum of opinions on what cognition should mean and how it manifests (e.g., Barton & Barrett, 2025; Bayne et al., 2019) does not imply a “lack of theoretical foundation” nor demand disqualification from the providing of commentary. Much of the critique brought forward by Jensen et al. (2025) concerns semantic and conceptual disagreements of this sort; they are of marginal relevance to studies which explored the utility of neuron count estimates to make palaeobiological predictions (Caspar et al., 2024; Herculano‐Houzel, 2023). In the case of Caspar et al. (2024), none of the conclusions drawn have been seriously challenged by Jensen et al. (2025).

Jensen et al. (2025) repeatedly suggest that Caspar et al. (2024) argued for the existence of particular cognitive similarities between crocodylians and Mesozoic dinosaurs, claiming that we postulated that *T. rex* in particular was “predominantly ‘crocodile‐like’”. Specific examples are not provided, which is unsurprising given that we did not make such statements. The focus of our argument was refutation of Herculano‐Houzel's view that large‐bodied theropods exhibited cognition and social behaviors akin to those of primates. We did not champion specific taxa as modern cognitive analogs and only employed reference to living crocodylians when in quest of neuroanatomical ground‐truthing with respect to estimates of endocranial fill (see Caspar et al., 2024 for a detailed justification). On this note, we also disagree with Jensen et al.'s (2025) claim that endocranial fill in endotherms (such as theropod dinosaurs) cannot be modeled via extrapolation from ectothermic lineages (“using a large ectothermic reptile as a model is unlikely to produce anything other than an ectothermic‐like estimate for dinosaurs”). No rationale for this is provided. Variation in endocranial fill among non‐avian sauropsids (from here on: reptiles) is pronounced and overlaps with that of mammals and birds (see, e.g., Allemand et al., 2017; Benoit, 2015; Knoll et al., 2024). Ancestral archosaurs exhibited a comparatively low degree of endocranial fill, a pattern conserved in crocodylians and—we assume, as seems prudent—various dinosaur lineages (Caspar et al., 2024). We are nevertheless aware that the accuracy of our brain size estimates is hard to gauge and that their interpretation certainly warrants some caution.

Indeed, many factors complicate efforts to reconstruct brain size, neuroanatomy, and cognition in Mesozoic dinosaurs, and we emphasized the uncertainty inherent to such efforts (a position echoed by Jensen et al., 2025). Hence, we avoided inferences about specific behaviors, the exception being tool‐use which we discussed with specific reference to claims made by Herculano‐Houzel (2023). We use the term “crocodile‐like cognition” (technically, this should admittedly have been crocodylian‐like) only once in our work to aggregate what we perceived as the consensus view in the literature prior to Herculano‐Houzel (2023). This perspective is neither idiosyncratic nor anachronistic: Hone (2024, p. 15), for example, recently summarized available evidence on Mesozoic dinosaur cognitive capacities in a similar but more casual way by stating “it is probably safe to say that on average, dinosaurs were […] approximately as intelligent as living crocodilians.” Jensen et al. (2025) seem to imply that cognitive comparisons between extant reptiles and non‐bird dinosaurs were abandoned in the wake of the dinosaur renaissance but this is simply not the case (e.g., Benton, 2023; Buchholtz, 2012; Hone, 2024; Hurlburt et al., 2013; Rogers, 1999; see also the continuing and widespread use of the Reptile Encephalization Quotient introduced by Hurlburt (1996) in works on non‐bird dinosaurs).

In line with this, we hold the position that it is perfectly justifiable to draw cognitive and behavioral comparisons between extant reptiles and Mesozoic dinosaurs as long as limitations are acknowledged. When considering overall endocast morphology and encephalization, most Mesozoic dinosaurs (including such non‐maniraptoriform theropods as *T. rex*) resemble extant reptiles more closely than they do their phylogenetically closer, but anatomically more derived, relatives the birds. In drawing attention to this, we do not equate these groups, but are highlighting their relative similarities, as a great number of researchers have done before us (e.g., Hopson, 1979; Hurlburt et al., 2013; Larsson, 2001; Morhardt, 2016; Rogers, 1999; Sampson & Witmer, 2007). Based on the same rationale, Jensen et al. (2023) argued that behavior in Mesozoic birds and “to an extent” that of more stem‐ward paravian dinosaurs can be reasonably modeled based on extant palaeognaths (but see, e.g., Gold & Watanabe, 2018 for some caveats). We want to make clear, however, that neuroanatomical similarities to reptiles do not preclude Mesozoic dinosaurs from exhibiting sophisticated behaviors commonly associated with endothermic vertebrates. Data on cognition in extant reptiles is patchy, yet evidence for complex social and communicative behavior, long‐distance navigational skills (at least in crocodylians and turtles), and performance overlaps with endothermic vertebrates in several cognitive domains all exist (Font et al., 2023; Grigg & Kirshner, 2015; Lin et al., 2021; Szabo et al., 2021; Wilkinson et al., 2025; note that Caspar et al., 2024 erroneously used the expression cognitive “dimension” instead of “domain”). Evidently, such behavioral feats are consistent with the presence of brains that are typically substantially smaller and lower in neuron count than those of birds (Kverková et al., 2022). In addition, recent studies indicate that various cognitive abilities comparable to those of large‐brained endotherms (incl. primates) are present in small‐brained actinopterygian fishes, another traditionally underestimated group of “lower” vertebrates (e.g., Agrillo & Bisazza, 2018; Kohda et al., 2022; Lucon‐Xiccato et al., 2017; Prétôt et al., 2016; Triki et al., 2025); further surprises may be in store, and we should be cautious when making claims about the cognitive inferiority of certain animal groups.

## 3 THE ENDOTHERMIC BRAIN HYPOTHESIS: ISSUES AND CHALLENGES

The EBH forms an important anchor point for the arguments presented by Jensen et al. (2025). It was published after the article by Caspar et al. (2024) and accordingly could not be incorporated into our discussion (Osvath et al., 2024). Note that numerous hypotheses discuss potential relationships between thermobiology, brain metrics, and cognition (see, e.g. Song et al., 2025) but that we will focus exclusively on the EBH as introduced by Osvath et al. (2024). Briefly, it states that tachymetabolic endothermic amniotes have evolved derived cognitive traits that enhance their abilities to find sufficient food to fuel their elevated metabolism (issues with assuming a dichotomy between ecto‐ and endotherms, especially in deep time, have been discussed by Caspar et al., 2024 and elsewhere). It is hypothesized that thermobiological and neurological traits to sustain foraging activities coevolved, eventually resulting in larger brain size, connectome alterations, and increased neuron densities in the telencephalon and cerebellum. The key cognitive difference between ecto‐ and endotherms, according to this hypothesis, concerns the ability within the latter to utilize a more elaborate “model‐based” mode of exploration and thus optimize foraging. Endotherms are suggested to explore their environment more efficiently by running more complex simulations in their minds (Osvath et al., 2024). For the sake of completeness, we want to mention that there was a recent reignition of the debate about whether non‐human mammals and birds actually exhibit model‐based cognition, which is immediately relevant for the EBH (Lind & Jon, 2025a, 2025b). So far, however, we certainly do not see a serious challenge to the idea of model‐based cognition in non‐human animals.

To sum up, the EBH devises a potential roadmap for the evolution of derived brains in endothermic lineages, and we do not criticize its principal theoretical framework, which we regard as sound.

For us, it is important whether the evolution of tachymetabolic endothermy and the aforementioned neurocognitive features are indeed directly linked. If this were the case, we would need to find consistent evidence for the co‐emergence of these traits in the fossil record. It is a requirement that this is established before we can rely on the EBH to make plausible predictions about the behavior and neurology of extinct endotherms, such as *T. rex* as well as to justify a marked cognitive distinctiveness from ectotherms.

As Jensen et al. (2025) concede, it is unclear to what extent model‐based exploration and its associated capacities actually became elaborated independently in extant birds and mammals or their endothermic ancestors. In any case, it remains obscure which neuroanatomical traits could determine the abilities hypothesized to be exclusive for endotherms and how (if possible at all) to identify them in fossil endocasts. In fossil taxa, a series of gradual, transitional configurations toward the modern avian and mammalian conditions can be observed. This morphological spectrum invites the arbitrary selection of traits that one might view as indicative of critical neurocognitive innovations (e.g., the modestly enlarged pallium of tyrannosaurids that Jensen et al. (2025) put much emphasis on). Assumptions about the functional significance of such traits will typically remain non‐falsifiable.

Jensen et al. (2025) cite Kverková et al. (2022) as evidence that increases in brain size and neuron density in the lineages culminating in birds and mammals coincided with the evolution of endothermy. This is misleading, because these data, important as they are for our understanding of amniote brain evolution, are obviously restricted to extant forms and have no bearing on ancient fossil taxa. They do not allow us to date the emergence of these characteristics beyond the emergence of the respective crown groups and do not provide any evidence for a coevolution of endothermy with said neurological traits. As argued previously (Caspar et al., 2024), we note that even among extant vertebrates, high neuron densities are not exclusive to tachymetabolic endotherms but can be found in various ectothermic actinopterygian fishes (Marhounová et al., 2019; compare Estienne et al., 2024). Marked changes in neuron density have also occurred within endothermic taxa without being accompanied by notable thermobiological shifts (Kverková et al., 2022).The evolution of vertebrate neuron densities appears complex and a close association with thermometabolism doubtful.

It is very difficult to test predictions of the EBH in fossil lineages outside of specific crown groups and, thus, to have informed opinions on how the EBH might apply to them. The only measurable traits relevant to the EBH in fossil taxa concern changes in brain size and proportions, and these, of course, must be derived from endocasts. Interestingly, the endocasts of many fossil groups for which tachymetabolic endothermy is hypothesized do *not* suggest that significant changes in neuroanatomy co‐emerged with elevated metabolic rates (see Table 1). Enlargement of the (fore)brain occurs much later in their respective histories, or not at all. Quantitative analyses have to further inspect these patterns but, for the time being, no reliable association is obvious. Neuroanatomical traits are, thus, not commonly considered proxies for tachymetabolic endothermy in fossil taxa (Grigg et al., 2022).

**TABLE 1:** Non‐exhaustive summary of evidence for whole‐body tachymetabolic endothermy in various fossil amniotes with general notes on endocast morphology. The majority of groups do not conform to predictions of the endothermic brain hypothesis. Note that endothermy might have evolved independently twice within Dicynodontia and was probably present in the common ancestor of dinosaurs and pterosaurs, if not already in the common ancestor of all archosaurs (see, e.g., Grigg et al., 2022). Specific limitations for each thermometabolic proxy need to be considered (see, e.g., Baumgart et al., 2025), and different proxies may suggest divergent estimates for the time of emergence of an endothermic metabolism (e.g., Bolton et al., 2025). Maniraptoriform dinosaurs and probainognathian (including mammaliaform) synapsids are not included. Regarding shifts in endocast shape, we only point out substantial departures from the plesiomorphic linearly arranged and roughly tubular endocast morphology of early amniotes that appear analogous to what is seen in modern mammals and birds. NA : Not applicable. Superscript numbers refer to references cited in the respective column.

Group	Lines of evidence for tachymetabolic endothermy	Earliest evidence for tachymetabolic endothermy in the given lineage	Endocranial evidence for pronounced shifts in relative brain size and/or shape analogous to extant endotherms	Time of emergence of substantial shifts in relative brain size	Selected references
Theropoda (excluding Maniraptoriformes)	Isotopic profiles of eggshells,^1^ bone histology,^2^ limb and body posture and associated energetic demands (in some forms),^3^ lipoxidation biomarkers,^4^ presence of insulating integumentary structures,^5^ biogeography^6^	Late Triassic (Carnian) ^2^(but note that endothermy might well have been present in the ancestral archosaur)	Not evident, only modest expansion of forebrain hemispheres (and more controversially the cerebellum) observable in basal coelurosaurs such as *Tyrannosaurus rex*. Low endocranial fill complicates reconstructions of brain (region) size	NA	^1^Laskar et al. (2020), ^2^Curry Rogers et al. (2024), ^3^Pontzer et al. (2009),^4^Wiemann et al. (2022), ^5^Campione et al. (2020), ^6^Chiarenza et al. (2024); endocast morphology reviewed by Paulina‐Carabajal et al. (2023)
Ornithischia	Isotopic profiles of eggshells,^1^ bone histology,^2^ limb and body posture and associated energetic demands (in some forms),^3^ presence of insulating integumentary structures,^4^ biogeography^5^	Early Jurassic (Hettangian)^2^(but note that endothermy might well have been present in the ancestral archosaur)	Not evident, only modest expansion of forebrain hemispheres observable in ornithopods such as hadrosaurs. Low endocranial fill in most groups complicates reconstructions of brain (region) size	NA	^1^Dawson et al. (2020), ^2^Legendre et al. (2016), ^3^Pontzer et al. (2009), ^4^Campione et al. (2020), ^5^Chiarenza et al. (2024); endocast morphology reviewed by Paulina‐Carabajal et al. (2023)
Sauropodomorpha	Isotopic profiles of eggshells,^1^ bone histology,^2,3^ lipoxidation biomarkers^4^	Late Triassic (Carnian) ^2^(but note that endothermy might well have been present in the ancestral archosaur)	Not evident, low endocranial fill complicates reconstructions of brain (region) size	NA	^1^Laskar et al. (2020), ^2^Curry Rogers et al. (2024), ^3^Legendre et al. (2016), ^4^Wiemann et al. (2022); endocast morphology reviewed by Paulina‐Carabajal et al. (2023)
Pterosauria	^1^Bone histology, ^2^presence of insulating integumentary structures. ^3^Lipoxidation end‐products in fossil bone, presence of powered flight	Late Triassic (Norian) ^2^(but note that endothermy might well have been present in the ancestral archosaur)	Yes, overall increase in brain size and endocranial fill is evident; with marked size increase of the forebrain and cerebellum. However, body mass estimates are challenging in this group, complicating the reconstruction of relative brain size (compare, e.g., Hurlburt, 1996; Witmer et al., 2003)	At least Early Jurassic, with the emergence of the clade Breviquartossa (compare Codorniú et al., 2016)	^1^Steel (2008), ^2^Campione et al. (2020), ^3^Wiemann et al. (2022); endocast morphology was discussed by Witmer et al. (2003) and Codorniú et al. (2016)
Ichthyosauria	^1^Bone histology, ^2^isotopic profiles from bone, ^3^biogeography (especially of early forms), ^4^thermobiological modeling	Early Triassic (Olenekian)^3^	Not evident. Apparent expansion of the cerebellum, at least in Early Jurassic forms, forebrain and overall endocast size/shape appear unexceptional, low endocranial fill and only partially ossified braincases complicate reconstructions of brain (region) size	NA	^1^Houssaye et al. (2014), ^2^Séon et al. (2025), ^3^Delsett et al. (2025), ^4^Brice and Grigg (2023); endocast morphology was discussed by Marek et al. (2015)
Pistosauria (including Plesiosauria)	^1^Bone histology, ^2^lipoxidation biomarkers, ^3^isotopic profiles from bone, ^4^biogeography, ^5^thermobiological modeling	At least Middle Triassic (Anisian) based on data from *Pistosaurus longaevus* ^1^	Not evident. Apparent expansion of the cerebellum, at least in Late Cretaceous forms, forebrain and overall endocast size/shape appear unexceptional, low endocranial fill and only partially ossified braincases complicate reconstructions of brain (region) size.	NA	^1^Fleischle et al. (2018), ^2^Wiemann et al. (2022), ^3^Leuzinger et al. (2023), Séon et al. (2025), ^4^Zverkov et al. (2021), ^5^Brice & Grigg (2023); endocast morphology was discussed by Allemand et al. (2019)
Pseudosuchia	^1^Bone histology, ^2^anatomy of extant forms, ^3^limb and body posture and associated energetic demands in some fossil lineages, ^4^estimates of mean arterial blood pressure	Potentially Late Permian (Wuchiapingian ‐ note that endothermy might well have been present in the ancestral archosaur); trait secondarily lost, probably close to the Triassic‐Jurassic boundary (discussed by Faure‐Brac, 2025)	Not evident. Low endocranial fill complicates reconstructions of brain (region) size	NA	^1^Legendre et al. (2016), ^2^Grigg et al. (2022), ^3^Pontzer et al. (2009), ^4^Seymour (2023); endocast morphology was discussed by von Baczko et al. (2022) and evidence for endothermy was extensively reviewed by Faure‐Brac (2025)
Dicynodontia	^1^Bone histology, ^2^isotopic profiles from bone, ^3^respiratory turbinates, at least in *Kawingasaurus*	Late Permian (probably Wuchiapingian in the case of *Kawingasaurus* ^3^ and Changhsingian in the case of later‐branching taxa within Dicynodontia^2^)	Yes, but apparently restricted to *Kawingasaurus* and to some extent, other cistecephalids. In *Kawingasaurus*, increased encephalization and an expanded forebrain (including a superficially neocortex‐like structure) have been reported In diverse later‐branching dicynodonts, overall endocast size/shape appears unexceptional. Low endocranial fill complicates reconstructions of brain (region) size here	NA for most lineages, Wuchiapingian in case of *Kawingasaurus*	^1^Botha‐Brink and Angielczyk (2010) and Olivier et al. (2017), ^2^Rey et al. (2017), ^3^Laaß and Kaestner (2023); the endocast of *Kawingasaurus* was described by Laaß and Kaestner (2017), see Angielczyk et al. (2019), and George et al. (2024) for discussion of other dicynodonts
Cynodontia (excluding Probainognathia, which include Mammaliaforms)	^1^Bone histology, ^2^isotopic profiles of fossil bone, ^3^respiratory turbinates	At least by the Middle Triassic (Anisian),^1,2^ but might have been inherited by earlier therapsids	Not evident, increased brain size is observable among Probainognathia, with further encephalization occurring in Mammaliaformes during the Late Triassic. Low endocranial fill complicates reconstructions of brain (region) size	NA, marked neuroanatomical shifts are restricted to Probainognathia	^1^Botha and Chinsamy (2001), ^2^Rey et al. (2017), ^3^Hillenius (1994); see George et al. (2024) and Bolton et al. (2025) for discussion of cynodontian endocasts

To focus on one example relevant to the discussion on non‐bird theropods (Caspar et al., 2024; Herculano‐Houzel, 2023), several lines of evidence indicate that tachymetabolic endothermy was already present in the early theropods of the Triassic (e.g., Curry Rogers et al., 2024; Legendre et al., 2016; Wiemann et al., 2022). Yet over approximately 150 million years of evolution as endotherms, even Late Cretaceous members of such lineages as Ceratosauria retained small brains and a plesiomorphic endocast morphology (Caspar et al., 2024; Laskar et al., 2020; Sampson & Witmer, 2007). Based on its endocast, brain organization in the Maastrichtian abelisaurid ceratosaur *Majungasaurus* has been specifically suggested to be “probably much like that of an extant crocodilian” (Sampson & Witmer, 2007).

Considering available endocranial data, coelurosaurs, mammaliaforms, and probably pterosaurs appear to be the only major known taxa of tachymetabolic amniotes which experienced a marked increase in relative brain size (especially in the telencephalon and cerebellum). They seem to be exceptional and not the norm (Table 1; see Bolton et al., 2025 for details on brain evolution trends in mammaliaforms and their ancestors) and even for these groups, the emergence of endothermy seems to in part substantially predate relevant neuroanatomical shifts (note, however, that the robustness of many thermometabolic proxies remains controversial and that it is unclear how quickly fully realized tachymetabolic endothermy can and did evolve; compare, for example, Grigg et al., 2022 and Bolton et al., 2025). If tachymetabolic endothermy did not induce such notable changes in brain size and proportions in the various other, sometimes highly successful and long‐lived amniote lineages exhibiting this trait, why should we assume that thermobiology is central to understanding derived neurological traits in the mammalian and avian lineage? Future discussion of the EBH should acknowledge these critical issues.

Jensen et al. (2025) go further by speculating that the derived neurological traits of living birds and mammals might have arisen through a cognitive “arms race” between their stem lineages. The question of when this race started, paused, or ended, and which specific taxa it involved, remains unaddressed. In the ancestors of mammals, notable increases in brain size, associated with expansion of the cerebral hemispheres and the cerebellum, appeared in the Anisian in the early Middle Triassic (Bolton et al., 2025); in the bird stem lineage, analogous changes emerged piecemeal in coelurosaurs across the Jurassic and Cretaceous (e.g., Larsson et al., 2000; Voris et al., 2025), the earliest known members of which date to the Middle Jurassic. Fossil data for the proposed scenario or even a particularly close ecological association between said lineages are absent. We are not aware of any data on extant lineages suggestive of cognitive arms races resulting in reciprocal neurological changes analogous to those hypothesized by Jensen et al. (2025). The proposal is thus implausible to us. When it comes to the impact of these ideas on our work, neither the EBH nor the proposal of a “cognitive arms race” in the early Mesozoic requires us to revise assumptions on dinosaur brains and behavior presented by Caspar et al. (2024). The claim that increased (fore‐)brain size and elevated neuron densities are tightly coupled with tachymetabolic endothermy and hence should be assumed for Mesozoic dinosaurs is not well substantiated.

## 4 NEURON COUNTS ARE NOISY AND STILL UNDERSTUDIED PREDICTORS OF ANIMAL COGNITION

Jensen et al. (2025) dedicated a significant portion of their text to the notion that telencephalic neuron counts are the “best available brain‐based proxy” (whatever that means in practice) for cognitive performance in birds and mammals. Contrary to their claims, we did not “question the link between cognitive performance and pallial neuron counts,” but discussed whether this link is sufficiently robust to allow behavioral inference. Strikingly, both Caspar et al. (2024) and Jensen et al. (2025) suggested that this is not the case, quite simply because the correlation between neuron counts and cognitive performance is too noisy. Numerous other authors have argued, based on both empirical and conceptual grounds, that bare (forebrain) neuron counts might not be decisive predictors of animal cognition (e.g., Barron & Mourmourakis, 2024; Barton & Barrett, 2025; Güntürkün et al., 2017; Triki et al., 2025), especially when making comparisons between distantly related groups that vary greatly in body mass and brain organization. It was important for us to provide a nuanced discussion on this topic to adequately address the position adopted by Herculano‐Houzel (2023), since the argument therein is that species resembling one another in forebrain neuron counts should be expected to exhibit similar cognitive abilities. We want to make clear that we did not argue that neuron counts are unimportant in comparative cognition research—the position that Jensen et al. (2025) appear to think we adopted in our previous article—but that they are just one of many factors determining cognitive complexity (Caspar et al., 2024).

Caspar et al. (2024) were aware of evidence that (fore)brain neuron numbers are correlated with certain aspects of cognition in multi‐species datasets and cited such literature (see below). These data do not entail, however, that neuron counts are particularly helpful when it comes to reconstructing behavior in fossil animals. To us, the idea that cognitive abilities are simply “unlocked” in a teleological fashion by adding undefined forebrain neurons appears implausible and lacks justification. Jensen et al. (2025) seem to have suggested that when they stated that evidence for high telencephalic neuron counts (that is, notably higher than those of extant reptiles) in unspecified Mesozoic dinosaurs suggests the presence of “largely unknown neurocognitive functions approaching those seen in birds.”

We expect that large‐scale comparative studies will help to determine the contribution of (forebrain) neuron counts to cognitive performance in the future (see, e.g., ManyBirds Project, 2025). After all, robust brain‐wide neuron count data have only been available for a short time and for a limited number of taxa. Very few studies have directly utilized them as cognitive correlates so far. This makes it bold to claim that total forebrain neuron counts function as the best available cognitive predictors at this point, even when compared to much criticized measures such as relative brain size (which, dependent on the taxa considered, may more or less strongly reflect (forebrain) neuron counts). While we fully agree that the latter metric is problematic, it has nevertheless been shown to correlate with various cognitive traits, particularly in birds (e.g., Audet et al., 2023; Iwaniuk, 2017; Lefebvre et al., 2004). Indeed, it is relevant to this lack of data that Jensen et al. (2025) conflated absolute and relative brain size measures with (pallial) neuron counts, a practice they elsewhere leveled as a criticism directed at Caspar et al. (2024). Jensen et al. (2025) cited 16 articles that, via qualitative cross‐study comparisons, would “make clear” that close ties between total forebrain neuron numbers and cognition exist. Yet, none of these papers, which were concerned with various cognitive domains, consider neuron counts directly. Most discuss or emphasize relative rather than absolute neuroanatomical measures as cognitive predictors (e.g., Audet et al., 2023; Lefebvre et al., 2004), find an effect of both, or only report significant effects of cerebellar and not forebrain size on behavior (Day et al., 2005).

These data do not compel us to agree with Jensen et al. (2025), and the same studies could easily be viewed as supporting, for instance, the relative rather than the absolute number of neurons as the best available cognitive predictor in birds and mammals. To our knowledge, Sol et al. (2022) is the only study so far that did analyze links between neuron counts and cognition by relying on both empirically determined neuron numbers (e.g., Barron & Mourmourakis, 2024, used problematic methods to reconstruct neuron numbers for a considerable portion of their dataset) and phylogenetically‐informed statistics (which are missing from, e.g., Herculano‐Houzel, 2017). In line with some of the aforementioned evidence, the authors found that both relative and absolute metrics can be linked to cognition. Further, this work had a clearly defined scope, dealing with instances of avian feeding innovations in the wild, for which neuron counts are indeed significant predictors. Whether its findings can be generalized for other taxa or aspects of cognition needs to be shown.

Intraspecific studies might also be able to provide valuable further insights, because based on the proclaimed reasoning that more neurons must translate to better cognitive performance, one would expect notable within‐species effects of this variable. Evidence for this assumption has indeed been reported for lab lineages of guppies specifically bred for divergent brain sizes (*Poecilia reticulata*—see Marhounová et al., 2019 and references therein) but has failed to be recovered in lab mice (*Mus musculus*—Neves et al., 2020). In humans, evidence for a moderate influence is available based on neuroanatomical proxies for neuron numbers but it is not straightforward, with marked differences in effects between the sexes (Genç et al., 2019, and references therein). In other words, the data is equivocal.

While Jensen et al. (2025) state that their views simply result from a “better familiarity with the comparative cognitive literature,” we propose instead that researchers can arrive at different conclusions when confronted with the same, in this case rather noisy, data. We hence prefer to adopt a perhaps more conservative stance and agree with the position that even in the most extensively studied group with respect to cognition—primates—evidence for an outstanding contribution of raw telencephalic or whole brain neuron counts for cognitive performance remains ambiguous (reviewed by Fichtel & Sehner, 2025; in this context it should be noted that we never assumed cognitive parity between, e.g., lemurs and apes, an erroneous statement made about our work by Jensen et al., 2025).

Finally, we want to note that new data on the cortical neuron counts of the Northern minke whale (*Balaenoptera acutorostrata*) brain suggest that previous studies may have greatly overestimated telencephalic neuron numbers in cetaceans (Avelino‐de‐Souza et al., 2025). We have cited these numbers in our previous paper as evidence that whales exceed humans in cortical neuron numbers without displaying superior cognitive abilities. This might not be the case and we now retract this specific argument.

## 5 NEURON COUNTS EVOLVE IN TANDEM WITH BODY SIZE

**FIGURE 1:**
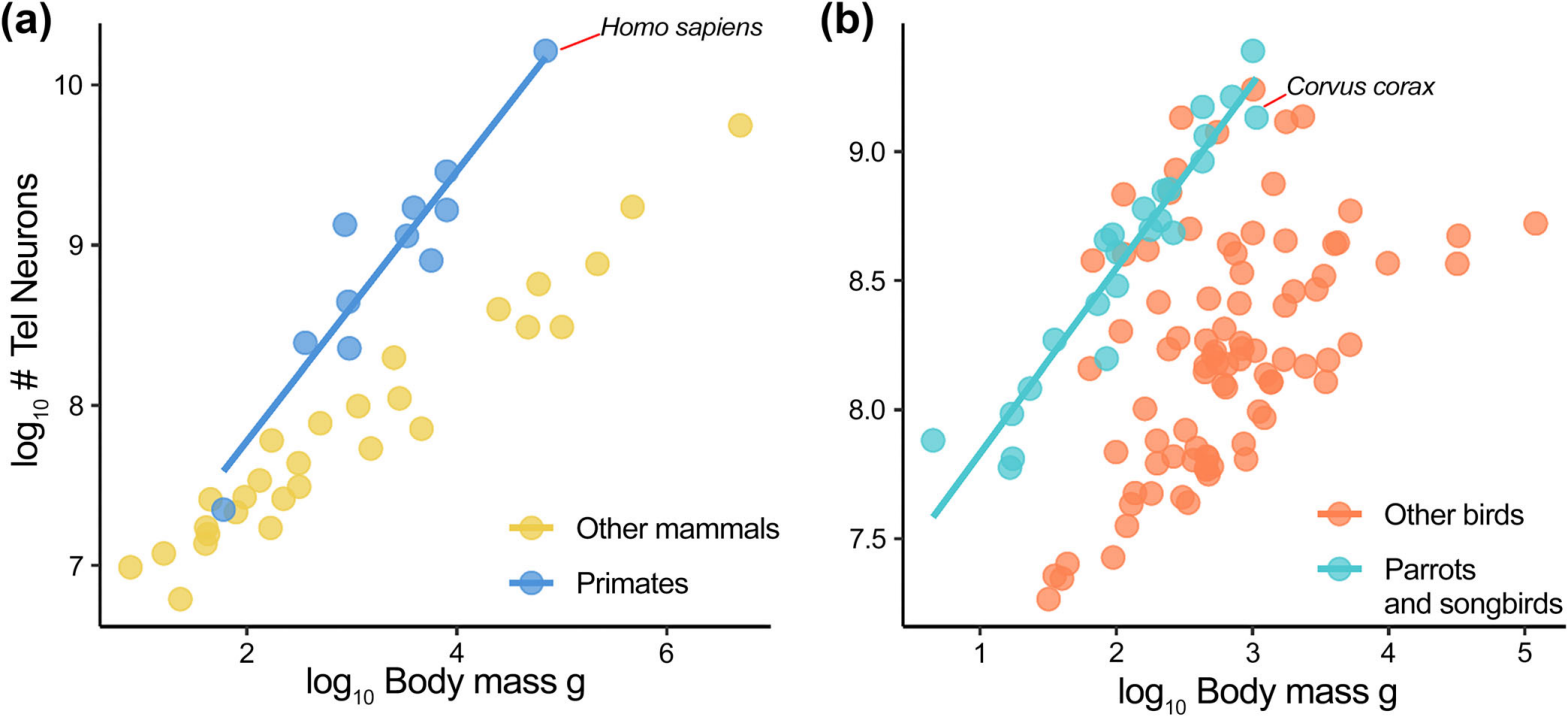
Log_10_ number of neurons in the telencephalon plotted against the log_10_ of body mass (g) in mammals (a) and birds (b). Shown are the regression lines for primates in (a) and for the species that belong to the clade Psittacopasserae (parrots and songbirds) in (b). Data taken from Herculano‐Houzel et al. (2015), Sol et al. (2022), and Kverková et al. (2022).

Jensen et al. (2025) state that “current evidence clearly suggests that telencephalic neuron count in amniotes does not scale with body size in a straightforward way.” This point is brought up to suggest that comparatively high neuron counts, like those estimated by Caspar et al. (2024) for *T. rex*, would not necessarily be expected in such large‐bodied animals. Previously, Herculano‐Houzel (2017, 2023) also argued that body mass is a poor correlate of forebrain neuron numbers and that the latter more closely reflects cognitive complexity. There is no question that neuron numbers in, for example, a telluravian bird vastly exceed those of a reptile of the same body mass. It is such comparisons that Jensen et al. (2025) and Herculano‐Houzel (2017, 2023) base their argument on. Nevertheless, although slopes can differ markedly between groups (e.g., Kocourek et al., 2025), we are unaware of any taxon in which neuron counts, like brain size overall, are not strongly predicted by body mass. In mammals—and using data from Herculano‐Houzel et al. (2015)—body size explains 78%–82% of variation in telencephalic neuron count data; for primates alone, this number is around 90% (Figure 1a; % obtained from R^2^s calculated using the *rr2* package (Ives & Li, 2018) in R, from the corresponding phylogenetic generalized least squares regression). Across birds (data taken from Sol et al., 2022, and Kverková et al., 2022), body size explains 60% of the variation in telencephalic neuron count; as in mammals, this value increases when more restricted clades are examined (Figure 1b). Among parrots and songbirds (Psittacopasserae), for example, about 88% of variation is explained by body mass. Importantly, no single species deviates substantially from the scaling rule of their particular clade: even famously large‐brained species like humans (*Homo sapiens*) and common ravens (*Corvus corax*) have the amount of neurons one would predict for their given body size. In line with the argumentation of Caspar et al. (2024), this suggests that comparatively high absolute neuron counts should be expected in such large‐bodied dinosaur species as *T. rex*. However, we cannot reasonably predict how different neuron number estimates conform to body mass estimates in these animals since neuron count scaling rules for them are simply unknown (as pointed out by both Caspar et al., 2024, and Jensen et al., 2025). In any case, the aforementioned patterns for extant species are puzzling if we assume that neuron counts do not, at least to some extent, reflect demands imposed on them by larger bodies. The specifics of these demands seem to vary considerably between clades and may relate to taxon‐specific sensory‐motor functions that scale with body mass and importantly involve the telencephalon (compare Caspar et al., 2024). If these two variables were truly unrelated, as Jensen et al. (2025) suggest, we would expect a lack of correlation between telencephalic neuron counts and body mass both between and within groups.

Claims that body size has negligible effects on brain size and neuron counts are also challenged by the observation that certain sexually dimorphic species exhibit marked differences in brain size and neuron counts (Cunha et al., 2022). As an extreme example, males in the Southern elephant seal (*Mirounga leonina*) have an average brain size of 1431 g, while that of females is only 899 g (Bininda‐Emonds, 2000).

## 6 COMMON GROUND: PROPOSAL FOR CONSENSUS

Despite the differences in opinions between Jensen et al. (2025) and ourselves, we emphasize our agreement on several issues pertinent to Mesozoic dinosaur cognition. It is unfortunate that Jensen et al. (2025) did not consider the substantial overlap that exists in our positions. In contrast to Herculano‐Houzel (2023), both Jensen et al. (2025) and Caspar et al. (2024) agreed that neuronal densities in fossil species cannot be reliably inferred based on relative brain size; in addition, both teams pointed out that endocranial fill in most Mesozoic dinosaurs cannot be accurately estimated based on extant comparisons, at least according to currently available methodology. Both teams also agree that telencephalic neuron count estimates for these animals remain exceedingly vague and are unsuitable for the inferring of cognitive complexity (though we note that Herculano‐Houzel, 2023 and Jensen et al., 2025 are seemingly more aligned with respect to the suitability of neuron counts as cognitive predictors in extant animals). Both teams view extant phylogenetic bracketing as applicable to the inferring of cognitive traits in extinct animals, and both agree that it can meaningfully contribute to our understanding of it. In applying it to Mesozoic dinosaurs, however, Caspar et al. (2024) raised a number of caveats (see also Hone, 2024) that remain, so far, unchallenged. This includes the secondary ectothermy of extant crocodylians, which might complicate their use in ectotherm‐endotherm comparisons. Perhaps most importantly, there is full agreement that multidisciplinary research combining evidence from fossils and the behavior of extant animals is needed to narrow down predictions about plausible behavior in fossil groups. Finally, both Jensen et al. (2025) and Caspar et al. (2024) exemplify how teams combining different expertise can move discussions in the field of comparative cognition.

## AUTHOR CONTRIBUTIONS

**Kai R. Caspar:** Conceptualization; writing – original draft; writing – review and editing; investigation. **Cristián Gutiérrez‐Ibáñez:** Conceptualization; visualization; formal analysis; investigation; writing – original draft; writing – review and editing. **Hady George:** Conceptualization; investigation; writing – original draft; writing – review and editing. **Thomas R. Holtz Jr.:** Conceptualization; investigation; writing – review and editing. **Darren Naish:** Conceptualization; investigation; writing – original draft; writing – review and editing. **Grant R. Hurlburt:** Conceptualization; investigation; writing – original draft; writing – review and editing.

## CONFLICT OF INTEREST STATEMENT

The authors declare no conflict of interest.

## Data Availability

The data that support the findings of this study are available from the authors upon reasonable request.
